# Collaboration between municipal and specialist public health care in tuberculosis screening in Norway

**DOI:** 10.1186/1472-6963-14-238

**Published:** 2014-05-27

**Authors:** Ingunn Harstad, Anne H Henriksen, Eli Sagvik

**Affiliations:** 1 Department of Pulmonary Medicine, St Olavs University Hospital, Po box, 3250, Sluppen, N-7006 Trondheim, Norway; 2 Department of Public Health and General Practice, Faculty of Medicine, Norwegian University of Science and Technology, NO 7489, Trondheim, Norway; 3 Department of Circulation and Medical Imaging, Faculty of Medicine, Norwegian University of Science and Technology, NO 7489, Trondheim, Norway; 4 City of Trondheim, Department of Infectious Disease Control, Municipality of Trondheim, Po Box 2300, Sluppen, 7004 Trondheim, Norway

**Keywords:** Tuberculosis, Screening, Asylum seekers, Refugees, Contact tracing, Collaboration

## Abstract

**Background:**

About 90% of new tuberculosis (TB) cases in Norway appear among immigrants from high incidence countries. There is a compulsory governmental tuberculosis screening programme for immigrants; immigrants with positive screening results are to be referred from municipal health care to the specialist health care for follow-up. Recent studies of the screening programme have shown inadequate follow-up. One of the main problems has been that patients referred for follow-up have not attended their appointment at the specialist health care.

TB screening in the municipality of Trondheim is done by two different teams: the Refugee Healthcare Centre (RHC) screens refugees and the Vaccination and Infection Control Office (VICO) screens all the other groups. Patients with positive findings on screening are referred to the hospital’s Pulmonary Out-patient Department (POPD). The municipal and referral level public health care initiated a project aiming to improve follow-up through closer collaboration.

**Methods:**

An intervention group and a pre-intervention control group were established for each screening group. During meetings between staff from the municipality and the POPD, inadequacies in the screening process were identified, and changes in procedures for summoning patients, and time and place for tests were implemented. For both the intervention group and the control group, time from referral until consultation at the POPD and number of patients that attended their first appointment were registered and compared.

**Results:**

In the VICO group, 97/134 (72%) of the controls and 109/123 (89%) of the intervention group attended their first appointment at the POPD after 30 weeks (median) and 10 weeks, respectively. In the RHC group 28/46 (61%) of the controls and 55/59 (93%) in the intervention group attended their first appointment after 15 and 8 weeks (median) respectively.

**Conclusion:**

Increased collaboration between the municipal and specialist health care can improve the follow-up of positive TB screening results.

## Background

For the year 2011, the World Health Organization (WHO) estimated a total of 8.7 million new (incident) and 12 million prevalent cases of tuberculosis (TB) worldwide [1]. Roughly estimated, one third of the world’s population has been infected with tuberculosis [2].

In Norway, the number of TB cases has increased in recent years and in 2011 The National TB Registry reported 361 cases, of which 88% were immigrants. Molecular studies have suggested that most cases of TB in Norway were due to imported strains and that about 80% of the cases were reactivation of previously contracted TB infection [3].

In Norway there is an extensive TB screening programme with the aim of preventing transmission of infection and development of disease in infected persons [4]. The guidelines from the National Institute of Public Health describe how, where and when different groups are to be screened [5]. Asylum seekers are screened on arrival in Oslo where cases suspected of active TB are diagnosed and treated. Asylum seekers not suspected of TB disease are transferred to other centres in Norway where the municipal public health care is to follow-up the screening results. Other groups, e.g. refugees, labour immigrants, students, family reunions, healthcare and childcare staff after returning from high incidence countries, and participants in contact tracing, are screened by the public health care in the municipality where they are living. The screening consists of a chest X-ray of everyone above the age of 15, and a Mantoux test of everyone up to the age of 40 except students and labour immigrants who are given a chest X-ray only [5]. If the Mantoux test is positive for a certain risk group an Interferon Gamma Release Assay (IGRA) test is performed before referral. When an abnormal X-ray, a Mantoux test 15 mm or a positive IGRA test is detected, the patient is referred to the local hospital, the department of pulmonary medicine, the department of pediatrics, or the department of infectious diseases. A specialist then conducts a full examination and diagnoses the patient with latent TB, active TB or any other diagnosis and decide whether to start treatment or not. Active TB is diagnosed by a combination of clinical examinations, tests for mycobacteria and radiology.

A previous study of the TB-screening programme for asylum seekers in Norway conducted in 2007 2009 showed that one third of patients with an abnormal X-ray on arrival were not followed up and only one third of persons with a positive Mantoux test were referred to a specialist for follow-up [6]. The study detected problems both in the municipal health care and in the specialist health care. The municipal health care often did not register the screening results or refer patients, and many referred patients did not attend their appointment at the hospital [7].

Many patients did not attend their appointment at the Pulmonary out-patient department (POPD) at St.Olavs Hospital either, and more than 400 patients with positive TB screening results had waited longer for an appointment than is acceptable according to governmental guidelines. This could possibly have led to unnecessary transmission of TB from undiagnosed cases, or serious complications because of late diagnosis and treatment. Others with latent TB could develop TB because they did not get prophylactic treatment. These concerns led to the planning of the present study.

The aim of this project was to improve the follow-up of patients with positive TB screening results through intervention that included increasing the collaboration between municipal and specialist public health care and new routines for summoning patients. To detect possible improvements, this study compared a group of patients after the intervention with a group from a time period before the intervention.

## Methods

### Study population and study sites

The municipality of Trondheim has 180 000 inhabitants and most immigrants in the county of Sør-Trøndelag (population: 298 000) live there. The city has a well-organized system for TB-screening and referral.

The public health care dealing with tuberculosis is organized in two different teams: the Vaccination and Infection Control Office (VICO) and the Refugee Healthcare Centre (RHC).

VICO is responsible for the compulsory screening for student and labour immigrants with their families, family reunions to Norwegians, health care workers or people working in child care after returning from high incidence countries, and contact tracing. VICO do Mantoux testing and refer for X-rays and IGRA tests in accordance with the guidelines [5]. In 2010, VICO screened 1600 persons for TB. The patients are informed about opening hours and there is no booking in advance.

The RHC do TB screening and offer primary health care for refugees and their families until they become included in the standard Norwegian health care system. For asylum seekers RHC offer follow-up of TB screening results and primary health care. The RHC examine about 200 persons each year and do Mantoux testing, refer for X-rays and IGRA tests and offer a general health examination including blood tests and HIV tests. Patients normally get an appointment within a week after arrival.

The POPD is part of the Clinic of Thoracic–and Occupational Medicine at St. Olavs University Hospital and is the only referral clinic for the County of Sør-Trøndelag for TB screening and pulmonary TB in adults. Patients screened for TB who are known to be HIV positive are referred directly to the Department of Infectious Diseases. Independent of diagnosis and country of origin, every patient is summoned by a general letter with information regarding the appointment, and if X-ray is needed a separate letter is sent from the Department of Radiology to the patient.

### Study design

This was a non-randomized study that compared a group of participants receiving an intervention with a similar group from the past with no intervention.

### Study end-points

Frequency of patients who attended their first consultation at the POPD, and the time from screening in the municipality to examination at the hospital by first call were registered end-points.

### Interventions

Regular meetings between staff at VICO, RHC, and the POPD were organized to map the screening process, locate the problems, discuss ideas for improvements, and test out the ideas. From the municipality of Trondheim, public health nurses and the infectious control physician participated, and from the POPD a nurse, the patient coordinators, the head of nursing and a chest physician (project leader) took part.

### Identified problems and interventions

During the project meetings, the whole screening process related to what was done where and when was described for each group. The two main problems identified were that many patients never arrived for their appointment at the POPD, and that there was a long delay between the primary screening and the time an appointment was given. For the patients who did not attend their appointments, the staff at VICO or RHC were asked to check if the patients had moved and this created extra work. For patients who had not attended their appointment at the POPD, a new appointment had to be given. This prolonged the waiting time at the POPD even more. Some patients even left the hospital after their X-rays or blood-tests without seeing the physician.

The main intervention was to change routines for summoning the patients. In addition to the summoning letters, a person contacted each of the patients by phone, either directly, through a contact person, or through a translator.

Another intervention was to change the timing of some of the tests to reduce the number of tests done at the hospital at the time of POPD appointment, and to reduce the total number of blood samples drawn For patients from RHC, all blood tests were done at the RHC; a new chest X-ray was also done prior to the appointment at the POPD. Thus, a separate summoning letter from the X-ray department was no longer needed. The patients from RHC were followed by a guide after they arrived at the hospital. The patients from VICO had an HIV test taken at the same time as they had their IGRA test.

### Inclusion criteria

**Figure 1:**
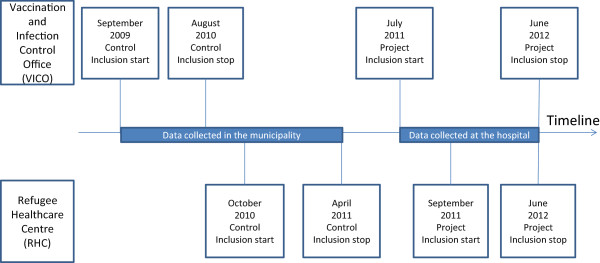
Timeline of patient inclusion.

All patients referred from VICO and RHC to the POPD in the study period were included (Figure 1).

### Data collection

Information about the control groups was collected retrospectively from paper files at VICO and computerized files at RFC (Figure 1). A designated nurse looked up all patients referred to the POPD in the actual time period and used information they had registered together with the information received from the POPD. When information from the POPD was not available in the municipal health files, this was collected from the hospital files.

Information about the intervention group was collected from the computerized hospital files. Referral lists from the municipality were used to identify the patients. The registration was done by the project leader.

### Data registration and analysis

The data were first registered by patient names in an Excel data base. Afterwards all patient- identifying information was removed and the files transferred to SPSS for windows, version 18 (Chicago, IL, USA) for analysis.

Frequencies were analyzed with proportions and 95% confidence intervals. Medians were compared across independent groups by non-parametric test (Mann-Whitney test) using Median Test for k samples and P 0.05 was considered statistically significant.

### Approvals and permissions

The Regional Ethics Committee, Mid-Norway, confirmed that the study did not require ethical approval. As a service evaluation project permission was needed and given from the Head of the Department of Thoracic and Occupational medicine, St.Olavs Hospital, and from the City Executive for Health and Welfare Services, City of Trondheim.

Approvals to use patients` files and to keep a secure file at the computer system were given from the above authorities, and at the hospital it was approved by the Data Protection Official as well. Data files were made anonymous before they were transferred to SPSS for analysis and files form the municipality and the hospital were kept and analyzed separately.

## Results

### Results of the registrations

#### VICO

The control group at VICO was screened between September 2009 and August 2010 and included 134 persons (Figure 1). The project period lasted from July 2011 to June 2012 and included 123 persons (Table 1). The control and the intervention groups had overlapping 95% confidence intervals (CI) for demographics, screening tests results, and cases with planned treatment for latent TB (Table 1). A higher percent diagnosed with latent TB was registered in the intervention group.

**Table 1:** Demographics and screening results for control and intervention patients according to place of screening

		**Vaccination and Infection Control Office (VICO)**		**Refugee Healthcare Centre (RHC)**	
**Period of registration**		**Control period Sept 2009-Aug 2010 (No 134)**	**Intervention period July 2011-June 2012 (No 123)**	**Control period Oct 2010-April 2011 (No 46)**	**Intervention period Sept 2011-June 2012 (No 59)**
Age	Median (range)	30 (16–74)	29 (19–77)	28,5 (17–59)	27 (16–71)
Gender	Female 95% CI	82 (61%) 53-69	86 (70%) 62-78	19 (41%) 27-56	29 (49%) 36-62
Mantoux test	>15 mm 95% CI	97 (72%) 65-80	105 (85%) 79-92	27 (59%) 44-73	36 (61%) 49-73
QuantiFERON	Positiv 95% CI	89 (66%) 58-74	74 (60%) 52-69	37 (80%) 69-92	42 (71%) 60-83
	Negativ 95% CI	41 (31%) 23-38	39 (32%) 23-40	8 (17%) 6-28	10 (17%) 7-27
Chest X-ray	Abnormal 95% CI	19 (14%) 8-20	28 (23%) 15-30	1 (2%) âˆ’2-6	9 (15%) 6-24
Diagnose	TB disease 95% CI	0	1 (1%) 0-2	1 (2%) 0-6	2 (3%) 0-8
	Latent TB 95% CI	44 (33%) 25-41	62 (50%) 42-59	28 (61%) 47-75	40 (68%) 56-80
	Previous TB 95% CI	4 (3%) 0-6	10 (8%) 3-13	0	6 (10%) 2-18
Treatment for latent TB	Yes 95% CI	19 (14%) 8-20	21 (17%) 10-24	16 (35%) 21-49	31 (53%) 40-65
	No 95% CI	90 (67%) 59-75	91 (73%) 66-82	7 (15%) 5-26	19 (32%) 20-44

In the control group, the most common reasons for screening were contact tracing (30), family reunion to Norwegian citizens (24), family reunion to students (23) and labour immigrants (19). In the intervention period, the reasons for screening were family reunion to Norwegian citizens (23), contact tracing (16), labour immigrants (28), family reunion to labour immigrants (15), and students (13).

In the control group, the patients came from 49 different countries and the most frequent countries were Norway (30), The Philippines (11) and China (10). In the intervention group, the patients came from 42 different countries and the most frequent were The Philippines (20), Norway (15) and Vietnam (8).

#### RHC

At the RHC, the control group was screened from October 2010 to April 2011 and included 46 persons: the intervention period was from September 2011 to June 2012, and 59 persons were included (Figure 1). A comparison of 95% CIs showed no differences between the control and the intervention group regarding demographic variables, screening test results, or numbers diagnosed with latent and active TB (Table 1). No cases of previous TB were registered in the control period but six were registered in the intervention period. Planned treatment for latent TB was equal in the groups. The persons screened at the RHC were asylum seekers, refugees or family reunion to refugees, but the available data was not sufficient to make it possible to differentiate between these groups.

In the control group, the patients came from 15 different countries and the most common countries of origin were Eritrea (12), Somalia (10), Liberia (4) and Ethiopia (3). The patients in the intervention group came from 12 different countries and the most frequent countries were Somalia (20), Ethiopia (8), Afghanistan (6), Eritrea (6) and Myanmar (6).

### Study end-points

The frequency of patients from VICO who attended the first consultation at the POPD increased from 97/134 (72%) in the control group to 109/123 (89%) patients in the intervention group (Table 2). The frequency among RHC patients increased from 28/46 (61%) to 55/59 (93%) patients. The CIs were not overlapping for either group. In the VICO group, time from screening in the municipality to examination at the hospital (by first call) was reduced from median 30 to median 10 weeks and this was a significant difference (p < 0.001) In the RHC group, the time was reduced from median 15 to median 8 weeks (p = 0.04). For the patients from RHC there were no differences in the total attendance to the POPD between the control and the intervention group (Table 2) but the control group needed to be summoned several times before they arrived.

**Table 2:** Attendance in specialist care related to place of primary examination

	**Vaccination and Infection Control Office (VICO)**		**Refugee Healthcare Centre (RHC)**	
	**Control period (No 134)**	**Intervention period (No 123)**	**Control period (No 46)**	**Intervention period (No 59)**
Attendance at hospital for 1st consultation	97 (72%)	109 (89%)	28 (61%)	55 (93%)
	(65–80) 95% CI	(83–94) 95% CI	(47–75) 95% CI	(87–100) 95% CI
Final attendance	115 (86%)	115 (93%)	44 (96%)	58 (98%)
	(80–92) 95% CI	(89–98) 95% CI	(90–100) 95% CI	(95–100) 95% CI
Time to attendance for 1st hospital consultation (weeks)	Median 30	Median 10	Median 15	Median 8
	(1–67) range	(2–57) range	(4–31) range	(0–41) range

## Discussion

Increased collaboration between public municipal health care and the POPD increased the numbers of patients attending their first call to the POPD and reduced the time from examination in the municipality to examination at the POPD.

The contents of screening programmes for immigrants show great variety among countries [8]. Studies from Switzerland and Canada have shown similar problems as in our study with follow-up of TB screening results among immigrants [9,10]. In more recent studies from Canada up to 50% of immigrants had adhered to post immigrant TB surveillance [11].

How can the follow-up of screening results be improved? A comparable study from USA showed improvement in the follow-up after arrival of people with abnormal results at their pre-entry screening. Without interventions, 25% of the immigrants arrived for follow-up. Step-wise intervention started with summoning letters, phone-calls, and finally home-visits and altogether 97.5% of the immigrants had their follow-up [12]. A review of migrant tuberculosis screening in Europe emphasized the importance of a good follow-up system and continuum of care. The review concluded that TB care should be integrated with general healthcare within a holistic approach [13]. One of the conclusions of a study about best practices in delivery of health care services to immigrants in Denmark was the need for collaboration between the different levels of health care and between the health care and the social sector [14].

As the intervention measures of our project were initiated simultaneously, it is hard to say which had most impact. Shorter time from examination in the municipality to summoning for the follow-up probably increased the numbers that arrived for examination, both because the patients remembered why they were referred, and fewer had moved to another municipality or changed addresses. Another reason for more patients turning up and shorter waiting time could be that the project led to increased awareness among staff at all levels. Because more patients attended their first appointment, fewer patients had to be summoned for a second or third time. This would reduce the workload and waiting lists at the POPD and as a consequence reduce the time before appointments.

The summoning process is another important issue, as was shown in the study from USA [12]. When only a letter is sent to the patient, no one knows whether the letter has been received, whether the address is correct or the patient has moved on, or whether the patient understands what the letter is all about. By contacting each patient directly or through an interpreter or a contact person, the contact was established and the message probably understood. It is possible that simplification of the summoning letter also helped; it was not necessary to send out extra letters from the X-ray department. The summoning letters could be improved further e.g. by further simplification and by translation into different languages.

Fewer patients were missed after they arrived at the hospital in the intervention period. X-rays and blood testing done before the appointment and a guide who received the patients from RHC when they arrived at the hospital could be reasons for that.

In the VICO group, there were more patients diagnosed with latent TB in the intervention group than in the control group. The reasons could be different interpretations of patient records or a real difference between the groups. Overall, more of the RHC patients were offered treatment for latent TB than in the VICO group. This is not adjusted for other factors, and it is beyond the focus of this paper to explain these results. Still, the results seem reasonable because refugees have higher risk for reactivation of TB than estimated risk in their home countries and e.g. labour immigrants or students from the same country [15,16].

### Possible consequences of the project

Because patients with abnormal findings at screening turned up sooner at the POPD, some patients were diagnosed with TB disease before having symptoms. As a consequence, they were less likely to infect so many others and the contact tracing would be less extensive. Others were possibly prevented from developing TB by getting preventive treatment at an earlier stage. Because fewer patients needed to be summoned a second or third time to the POPD, the waiting time and waiting lists where shortened. This benefited both TB screening patients and other patients attending the POPD. In the municipality, the requests for checking up missing people were considerably reduced and this reduced the total workload. Performing screening in the municipality was probably more rewarding when a conclusion followed quickly?

Can the results of this project be used elsewhere? Local differences in the organization of the screening process make it difficult to use the experiences from our project directly. But the process of closer collaboration between different levels of health care dealing with screening could be transferred everywhere. A thorough mapping of who is doing what and where in the screening process, and what the main problems are locally, is a useful way of starting the process of improvement. Our results indicate that this way of dealing with problems between health care levels could also be used for other specific patients groups that have problems using the ordinary health care system.

### Strengths and limitations

The project was started to improve the follow-up of positive screening tests, not to evaluate the screening programme itself. Patients with alarming symptoms or grossly abnormal X-rays were fast tracked through the system and not registered in this project. The yield of screening can thus not be evaluated.

The control groups were registered in the municipality from their files by a nurse and the project groups from the hospital files by the project leader. When no information from the hospital was received in the municipality, the patient information was checked in the hospital files for more complete information. This would make the data collection and registration as similar as possible for the control and project groups. Still some data could be missing or registered in a different way at different levels.

From one year to the next, countries of origin of the screened persons differ and so do the numbers in each group. There were even some patients from Norway included who were screened according to the regulations. All these factors could influence the degree to which the patients would attend their appointment.

The strength of the study is the simplicity, and how easily the information can be used in other settings. The project is close to ordinary routines and can be handled in practice with minor adjustments.

## Conclusion

A project of increased collaboration between the municipal public health care and POPD in the follow-up of TB screening increased the numbers that attended their first appointment in the POPD, and decreased the time from examination in the municipality until the POPD examination. As a result, some cases of active TB were detected before they had any symptoms, the work-load and waiting lists at the POPD were reduced and the municipality got fewer enquiries about missing persons.

## Abbreviations

TB: Tuberculosis; RHC: Refugee healthcare centre; VICO: Vaccination and infection control office; POPD: Pulmonary out-patient department; IGRA: Interferon Gamma Release Assay; HIV: Human immune deficiency virus; WHO: World Health Organization; CI: Confidence interval.

## Competing interests

All authors declared no competing interests.

## Authors’ contributions

IH: was the project leader throughout the study, participated in the planning, carried out the hospital part of the data collection, did the statistical analysis and drafted the manuscript. AHH: participated in the planning and running of the study, gave important input into the manuscript. ES: participated in the planning of the study, was responsible for the municipality’s part of running the study and gave input into the manuscript. All authors read and approved the final manuscript.

## Pre-publication history

The pre-publication history for this paper can be accessed here:

http://www.biomedcentral.com/1472-6963/14/238/prepub

## References

[B1] WHO Global TB Report 2011 http://www.who.int/tb/publications/global_report/2011/gtbr11_full.pdf

[B2] Lonnroth KCK Chakaya JM Chauhan LS Floyd K Glaziou P Raviglione MC Tuberculosis control and elimination 2010–50: cure, care and social development Lancet 2010 14 1814 1829 10.1016/S0140-6736(10)60483-7 20488524

[B3] Dahle UR Eldholm V Winje BA Mannsaker T Heldal E Impact of immigration on the molecular epidemiology of Mycobacterium tuberculosis in a low-incidence country Am J Respir Crit Care Med 2007 14 9 930 935 10.1164/rccm.200702-187OC 17673698

[B4] Forskrift om tuberkulosekontroll 2009 http://lovdata.no/dokument/SF/forskrift/2009-02-13-205

[B5] Folkehelseinstituttet Tuberkuloseveilederen 2010 Oslo: Folkehelseinstituttet

[B6] Harstad I Heldal E Steinshamn SL Garasen H Jacobsen GW Tuberculosis screening and follow-up of asylum seekers in Norway. A cohort study BMC Public Health 2 2009 14 1 141 10.1186/1471-2458-9-141 PMC2689201 19442260

[B7] Harstad I Heldal E Steinshamn SL Garasen H Winje BA Jacobsen GW Screening and treatment of latent tuberculosis in a cohort of asylum seekers in Norway Scand J Public Health 2010 14 3 275 282 10.1177/1403494809353823 19914972

[B8] Alvarez GG GB Rumman KA Altpeter E Chemtob D Douglas P Erkens C Helbling P Hamilton I Jones J Matteelli A Paty MC Posey DL Sagebiel D Slump E Tegnell A Valin ER Winje BA Ellis E A comparative examination of tuberculosis immigration medical screening programs from selected countries with high immigration and low tuberculosis incidence rates BMC Infectious Diseases 2011 14 3 10.1186/1471-2334-11-3 PMC3022715 21205318

[B9] Breuss E Helbling P Altpeter E Zellweger J-P Screening and treatment for latent tuberculosis infection among asylum seekers entering Switzerland Swiss med wkly 2002 14 197 200 12070785 10.4414/smw.2002.09901

[B10] Richards B Kozak R Brassard P Menzies D Schwartzman K Tuberculosis surveillance among new immigrants in Montreal Int J Tuberc Lung Dis 2005 14 8 858 864 16104631

[B11] REE L The Canadian Tuberculosis Standards 2007 vol. 2012. http://www.respiratoryguidelines.ca/tb-standards-2013

[B12] Catlos EKCM Bhatia G Gedin S Lewis J Mohle-Boetani JC Public Health Interventions to Encourage TB Class A/B1/B2 Immigrants to Present for TB Screening Am J Respir Crit Care Med 1998 14 1037 1041 10.1164/ajrccm.158.4.9801024 9769257

[B13] Klinkenberg E Manissero D Semenza JC Verver S Migrant tuberculosis screening in the EU/EEA: yield, coverage and limitations Eur Respir J 2009 14 5 1180 1189 10.1183/09031936.00038009 19880618

[B14] Jensen NKNS Krasnik A Expert opinion on "best practices" in the delivery of health care services to immigrants in Denmark Dan Med Bull 2010 14 8 1 5 20682134

[B15] Hadzibegovic DS Maloney SA Cookson ST Oladele A Determining TB rates and TB case burden for refugees Int J Tuberc Lung Dis 2005 14 4 409 414 15830746

[B16] Lobato MN Mohamed MH Hadler JL Tuberculosis in a low-incidence US area: local consequences of global disruptions Int J Tuberc Lung Dis 2008 14 5 506 512 18419885

